# Transcriptome Analysis of LLC-PK Cells Single or Coinfected with Porcine Epidemic Diarrhea Virus and Porcine Deltacoronavirus

**DOI:** 10.3390/v16010074

**Published:** 2023-12-31

**Authors:** Yanzhen Lu, Ruiming Yu, Lixin Tong, Liping Zhang, Zhongwang Zhang, Li Pan, Yonglu Wang, Huichen Guo, Yonghao Hu, Xinsheng Liu

**Affiliations:** 1 College of Veterinary Medicine, Gansu Agricultural University, Lanzhou 730070, China; luyanzhen0914@126.com (Y.L.);; 2 State Key Laboratory for Animal Disease Control and Prevention, OIE/National Foot-and-Mouth Disease Reference Laboratory, Key Laboratory of Animal Virology of Ministry of Agriculture, Lanzhou Veterinary Research Institute, Chinese Academy of Agricultural Sciences, Lanzhou 730046, Chinapanli@caas.cn (L.P.);

**Keywords:** porcine epidemic diarrhea virus (PEDV), porcine deltacoronavirus (PDCoV), coinfection, differential transcriptomics, interferon-stimulated genes (ISGs)

## Abstract

Porcine epidemic diarrhea virus (PEDV) and porcine deltacoronavirus (PDCoV) are the two most prevalent swine enteric coronaviruses worldwide. They commonly cause natural coinfections, which worsen as the disease progresses and cause increased mortality in piglets. To better understand the transcriptomic changes after PEDV and PDCoV coinfection, we compared LLC porcine kidney (LLC-PK) cells infected with PEDV and/or PDCoV and evaluated the differential expression of genes by transcriptomic analysis and real-time qPCR. The antiviral efficacy of interferon-stimulated gene 20 (ISG20) against PDCoV and PEDV infections was also assessed. Differentially expressed genes (DEGs) were detected in PEDV-, PDCoV-, and PEDV + PDCoV-infected cells at 6, 12, and 24 h post-infection (hpi), and at 24 hpi, the number of DEGs was the highest. Furthermore, changes in the expression of interferons, which are mainly related to apoptosis and activation of the host innate immune pathway, were found in the PEDV and PDCoV infection and coinfection groups. Additionally, 43 ISGs, including GBP2, IRF1, ISG20, and IFIT2, were upregulated during PEDV or PDCoV infection. Furthermore, we found that ISG20 significantly inhibited PEDV and PDCoV infection in LLC-PK cells. The transcriptomic profiles of cells coinfected with PEDV and PDCoV were reported, providing reference data for understanding the host response to PEDV and PDCoV coinfection.

## 1. Introduction

Both PEDV and PDCoV are enclosed, single-stranded RNA viruses that are members of the same order (Nidovirales) and family (Coronaviridae) but members of different genera [1]. The clinical symptoms caused by these swine coronaviruses are similar and indistinguishable and include severe diarrhea, vomiting, dehydration, and weight loss [2]. PEDV and PDCoV have different genome structures. The genome of PEDV is approximately 28 kb in length and consists of the open reading frames ORF1a and ORF1b, spike (S), ORF3, envelope (E), membrane (M), and nucleocapsid (N), which are flanked by 5′- and 3′-untranslated regions (UTRs) [3]. In contrast, the genome of PDCoV is approximately 25 kb in length and comprises ORF1a/1b, S, E, M, N, nonstructural protein 6 (Nsp6), and Nsp7, which are flanked by 5′- and 3′-UTRs [4]. Although their genome structures are different, the spike proteins they encode play important roles in pathogenesis. The spike protein contains two domains, S1 and S2. S1 binds to the cell receptor, and S2 fuses with the cell membrane [5,6].

PEDV is a highly contagious porcine enteric pathogen that can infect pigs of all ages [7]. In the UK, PEDV was originally identified in swine populations in 1971 and was first confirmed as the cause of PED in 1978 [8]. PEDV became ubiquitous once it emerged, particularly in Asia. At the end of 2010, a PEDV outbreak occurred in several pig-producing provinces in southern China [9]. Since then, the disease has spread throughout other provinces of China and has led to enormous economic losses within the pork industry. In 2013, PEDV was first reported in the USA, after which a highly pathogenic PEDV strain emerged and swept across the United States, leading to the loss of more than 10% of piglets [10]. In addition, a study reported data on the spread of coronavirus in pigs, indicating that the coronavirus can spread between wild boar populations and domestic pigs. In the pig population in southern Italy, PEDV is the most prevalent CoV. Disease transmission has been shown to be influenced by farm size and animal density, with domestic pig populations being more susceptible to the spread of these viruses than wild boar populations because of their high density [11,12]. The emergence and re-emergence of PEDV have caused severe economic losses and pose significant public health concerns worldwide.

Although the virus was originally reported in Hong Kong, China, in 2012, the etiological significance of PDCoV was not identified until 2014 [4,13]. In the US, PDCoV first became known as a major cause of diarrheal illness in swine herds in 2014 and later rapidly spread to several other states adjacent to Ohio [14,15]. Subsequently, PDCoV was rapidly detected in many countries, including Canada, Mexico, South Korea, China, Japan, Lao PDR, Vietnam, and Thailand, and poses a significant threat to the swine industry [16,17,18,19,20,21,22,23].

PDCoV causes lower mortality than PEDV in piglets [13,24]. Natural coinfections with swine enteric coronaviruses are common. Coinfection with PEDV and PDCoV causes massive economic losses in the pig industry globally. Previous studies have shown that coinfection with PEDV occurs in up to 50% of PDCoV-infected diarrheal pigs [25,26]. In South Korea, coinfection of PEDV with PDCoV was also predominant in collected diarrheal samples [17]. The coinfection rate of PEDV and PDCoV was high, reaching 60.4% in diarrheal pigs in Henan Province, China [25]. Furthermore, compared to a single infection, coinfection with PEDV and PDCoV in piglets could worsen clinical symptoms and have a synergetic effect on the intestinal antiviral immune response and the modulation of inflammatory cytokine expression [27,28,29]. In addition, coinfection of PEDV and PDCoV can alter PEDV tropism, which may affect the outcome of viral disease in piglets [30].

In recent years, transcriptomics has become an important means to study the response of host cells to virus infection, and this approach provides a new research direction for this study of viral pathogenesis. However, the cellular responses following infection with PEDV and/or PDCoV alone or in combination are poorly understood. Consequently, LLC porcine kidney (LLC-PK) cells were used in this study. These cells were either single-infected or coinfected with PEDV and/or PDCoV. Next, transcriptomic analysis and real-time qPCR were used to compare differentially expressed genes (DEGs), particularly interferon-stimulated genes (ISGs), between the groups. The antiviral efficacy of interferon-stimulated gene 20 (ISG20) against PDCoV and PEDV infections was also assessed.

## 2. Materials and Methods

### 2.1. Cells and Viruses

LLC-PK cells were purchased from the American Type Culture Collection (ATCC, CL-101) and used for viral culture and TCID_50_ assays of PEDV and PDCoV. A virulent Chinese GIIa PEDV strain, CH/HBXT/2018, was isolated in our laboratory from diarrheal samples of a suckling piglet suffering from acute diarrhea using Vero cells. The PDCoV strain CH/XJYN/2016 was isolated in our laboratory from the intestinal samples of a suckling piglet suffering from acute diarrhea utilizing LLC-PK cells. The LLC-PK cells were cultured in modified Eagle medium (MEM) (HyClone, Logan, UT, USA) supplemented with 10% fetal bovine serum (FBS; Gibco, Grand Island, NY, USA) and a penicillin–streptomycin mixture at 37 °C in a 5% CO_2_ environment.

### 2.2. Antibodies and Reagents

A mouse monoclonal antibody (mAb) against PEDV N and a mouse anti-PDCoV N mAb were prepared and stored in our laboratory [31,32]. Mouse anti-β-tubulin mAb and HRP-labeled goat anti-mouse IgG were purchased from Abmart (Shanghai, China). pCDNA3.1-ISG20-myc-his was constructed by our laboratory. We purchased TRIzol reagent and Lipo8000TM Transfection Reagent from Beyotime Biotechnology (Shanghai, China). Pierce ECL Western Blotting Substrate was obtained from Thermo Scientific (Waltham, MA, USA).

### 2.3. One-Step Growth Curve

LLC-PK cells were infected with PEDV (MOI = 0.01), PDCoV (MOI = 0.01), or PEDV and PDCoV (MOI = 0.01 for each virus), and the supernatant was collected at 3, 6, 12, 24, and 36 hpi. After the supernatant was collected, the 50% tissue culture infectious dose (TCID50) was assessed using the Reed–Muench technique. Briefly, LLC-PK cells were cultivated for 12 h at a density of 1 × 105 cells/well in 96-well tissue culture plates. This was followed by three washes with PBS. The obtained supernatant was diluted ten times (10^−1^ to 10^−10^) using a maintenance solution containing 20 µg/mL trypsin. Afterward, the cells were infected with a diluted virus for 3, 6, 12, 24, or 36 h at 37 °C in 5% CO_2_. An inverted microscope was used to monitor the CPE every day.

### 2.4. Immunofluorescence Assay (IFA)

After the LLC-PK cells were plated in 6-well plates, they were allowed to reach 70–80% confluency overnight. Afterward, the cells were infected with 20 μg/mL trypsin containing PEDV (MOI = 0.01), PDCoV (MOI = 0.01), or PEDV and PDCoV (MOI = 0.01 for each virus). At 6, 12, 18, and 24 h post-infection, the medium was removed, and the cells were washed three times with sterile PBS. The cells were then fixed with cold 4% paraformaldehyde for 60 min at 4 °C. Afterward, 1 mL of 0.5% Triton X-100 (Solarbio, Beijing, China) was added, and the cells were incubated for 10 min at room temperature. After three washes in sterile PBS, the cells were blocked for one hour using 5% bovine serum albumin (Solarbio, Beijing, China). After washing three times with sterile PBS, a monoclonal anti-PDCoV N protein antibody was used to detect PEDV- and PDCoV-infected cells. The cells were incubated with a rabbit polyclonal anti-PEDV N protein antibody (prepared and stored in our laboratory), Alexa Fluor^®^ 488-conjugated goat anti-mouse IgG, and Alexa Fluor^®^ 549-conjugated goat anti-rabbit IgG (Abcam, Cambridge, UK) at room temperature for 2 hpi. Cell nuclei were stained with 0.01% 4′,6-diamidino-2-phenylindole (DAPI) (Solarbio Life Science, Beijing, China). Using an inverted fluorescence microscope (Olympus, Tokyo, Japan), fluorescence images were captured.

### 2.5. Viral Infection

After the LLC-PK cells were plated in 6-well plates, they were allowed to reach 70–80% confluency overnight. The cells were then cultivated at 37 °C in 5% CO_2_ for 0, 6, 12, or 24 h after being inoculated with PEDV (MOI = 0.01), PDCoV (MOI = 0.01), or PEDV and PDCoV (MOI = 0.01 for each virus). Additionally, the cells were treated with 20 μg/mL trypsin. The cells were collected to be extracted and lysed to isolate RNA.

### 2.6. RNA Extraction

A TRIzol reagent was used to extract total RNA from virus- or sham-infected cells in accordance with the manufacturer’s instructions. Total RNA was dissolved in 60 µL of RNAse-free ddH_2_O and stored at −20 °C.

### 2.7. Real-Time Quantitative PCR

qPCR was conducted using a Step TB Green^®^ Prime Script™ RT–PCR Kit II on a Bio-Rad C1000 real-time PCR machine; β-actin was used as a housekeeping gene. The real-time PCR primers used are listed in Table 1. The RT–qPCR system consisted of 2× One Step TB Green RT–PCR Buffer (10 μL), Prime Script 1 Step Enzyme Mix (0.8 μL), upstream primer (0.8 μL), downstream primer (0.8 μL), template RNA (2 μL), and RNAse-Free ddH_2_O (5.6 μL). The reaction conditions were as follows: 42 °C for 5 min and 95 °C for 10 s; 40 cycles of 95 °C for 5 s and 60 °C for 20 s. Each sample was tested in triplicate. The 2^(−DDCt)^ method was used to determine the relative transcript levels of the target genes, which are displayed as fold changes in relation to the transcript level of the corresponding untreated control samples.

### 2.8. RNA-Seq Analysis

Total RNA was treated with RNase-free DNase I to prepare a cDNA library. Afterward, the mRNA was separated into fragments approximately 300 bp in length by ion interruption utilizing magnetic oligo (dT) beads for purification. Reverse transcriptase and 6-base random primers were used to create first-strand cDNA using RNA as a template, and the first-strand cDNA was subsequently used as a template to generate second-strand cDNA. RNA integrity was evaluated using an Agilent 2100 Bioanalyzer (Agilent Technologies, Santa Clara, CA, USA). Afterward, these libraries were sequenced on an Illumina NovaSeq platform (Illumina, San Diego, CA, USA) utilizing second-generation sequencing technology.

### 2.9. GO and KEGG Enrichment Analysis

Pathway analyses were performed using the Gene Ontology (GO) and Kyoto Encyclopedia of Genes and Genomes (KEGG) online tools, and the related pathways in the databases were subsequently matched to the annotated genes. Next, cluster analysis of the enriched terms was performed. Differentially expressed proteins (DEPs) were identified using the criterion of a 2-fold increase and a *p* value < 0.05 to ensure valid comparisons between samples. GO and KEGG pathway enrichment analyses were performed via STRING.

### 2.10. Overexpression or Knockdown of ISG20

LLC-PK cells were cultured in 6-well tissue culture plates until they reached 70∼80% confluence. Then, the cells were transfected with 1.5 μg of pCDNA3.1-ISG20-myc-his using Lipofectamine 8000 Transfection Reagent or 20 μM siRNA targeting ISG20 (si-ssc-ISG20) using Lipofectamine 8000 Transfection Reagent for 24 h.

### 2.11. Western Blotting

LLC-PK cells were harvested in lysis buffer containing a protease inhibitor and ultrasonicated, after which any insoluble components were removed by centrifugation. A BCA protein assay kit (Beyotime Biotechnology, Shanghai, China) was used to determine the overall protein concentration. The proteins were separated on 12% sodium dodecyl sulfate–polyacrylamide gel electrophoresis (SDS–PAGE) gels and then transferred onto nitrocellulose (NC) membranes, which were blocked in 5% nonfat milk. Afterward, the membranes were incubated with the primary antibody for two hours at room temperature or overnight at 4 °C. The membranes were washed three times with TBST before being incubated with HRP-labeled goat anti-mouse (or anti-rabbit) IgG (H + L) secondary antibodies (Beyotime Biotechnology) for one hour at room temperature. After the membranes were washed, Pierce ECL Western Blotting Substrate (Thermo Scientific) was used to visualize the protein bands.

### 2.12. Statistical Analysis

A Student’s *t* test was used for all the experimental analyses. The data from three independent qPCR runs are expressed as the means ± standard deviations (SDs) and were statistically analyzed by a Student’s *t* test using GraphPad Prism 8.0 software (USA). *p* values < 0.05 were considered to indicate statistical significance.

## 3. Results

### 3.1. Proliferation Kinetics of LLC-PK Cells Infected with PEDV, PDCoV, or PEDV and PDCoV

**Figure 1:**
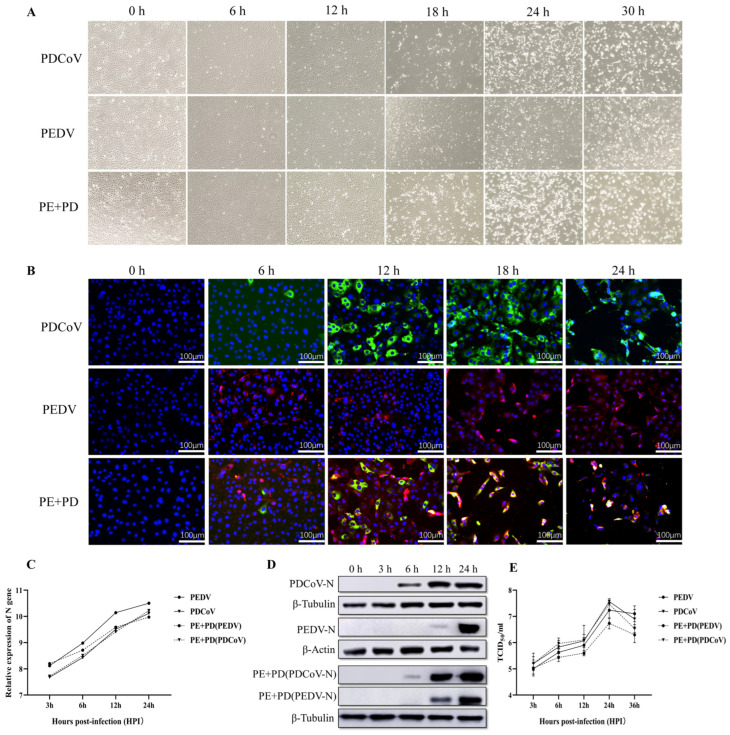
PEDV, PDCoV, or PEDV and PDCoV infection in LLC-PK cells. (**A**) Cytopathic effects (CPEs) in LLC-PK cells at 6, 12, 18, 24, and 30 hpi after PEDV, PDCoV, or PEDV and PDCoV infection; mock-infected cells were studied at 0 hpi as a negative control. (**B**) Immunofluorescence staining of infected LLC-PK cells at 6, 12, 18, and 24 h post-infection; mock-infected cells were examined at 0 hpi as a control. (**C**) qPCR analysis of LLC-PK cells infected with PEDV, PDCoV, or PEDV and PDCoV. LLC-PK cells were infected with PEDV (MOI = 0.01), PDCoV (MOI = 0.01), or PEDV and PDCoV (MOI = 0.01 for each virus). The cells were collected at 3, 6, 12, and 24 hpi, after which RT–PCR analysis was performed. (**D**) Virus replication in PEDV-, PDCoV-, or PEDV- and PDCoV-infected LLC-PK cells at 12 and 24 h post-infection was analyzed using Western blotting. (**E**) One-step growth curve of LLC-PK cells infected with PEDV, PDCoV, or PEDV and PDCoV. Viruses were collected from LLC-PK cells, followed by TCID50 evaluation. Abbreviations: PE + PD: PEDV + PDCoV.

Following virus infection, the levels of viral genes and viral titers were evaluated to determine the infectivity and propagation kinetics of PEDV, PDCoV, or PEDV and PDCoV in LLC-PK cells. At 0 hpi, no CPEs were detected in the infected cells, as shown in Figure 1A. In LLC-PK cells infected with PEDV, PDCoV, or PEDV and PDCoV, distinct CPEs were visible at 18 hpi. LLC-PK cells infected with PEDV were obviously enlarged and rounded, and they began to shed virus at 30 hpi. At 24 hpi, PDCoV caused significant host cell lysis, shedding, obvious enlargement, or membrane fusion, and almost all the cells were detached at 30 hpi. At 24 h after PEDV and PDCoV infection, almost all the LLC-PK cells had detached. Furthermore, according to the IFA data, at 6 h after PEDV, PDCoV, or PEDV and PDCoV infection, the viruses started to replicate, and this replication progressively increased by 24 hpi (Figure 1B). However, CPEs and IFA were significantly enhanced after co-infection with PEDV and PDCoV compared with PEDV or PDCoV alone.

As shown in Figure 1C,D, the qPCR results demonstrated that virus levels progressively increased in LLC-PK cells infected with PEDV, PDCoV, or PEDV and PDCoV. However, compared with a single infection of PEDV or PDCoV, the level of PEDV virus decreased significantly after co-infection, but the level of PDCoV virus did not change significantly (Figure 1C). According to the Western blot results, following inoculation with either PEDV or PDCoV alone, the PEDV nucleocapsid protein could be detected at 12 and 24 hpi, but the PDCoV nucleocapsid protein could be detected at 6, 12, or 24 hpi. In PEDV and PDCoV-coinfected LLC-PK cells, the PEDV nucleocapsid protein was found at 12 and 24 hpi, while the PDCoV nucleocapsid protein was found at 6, 12, and 24 hpi (Figure 1D). Additionally, in LLC-PK cells, the titer of PEDV alone or in combination with PDCoV was assessed. At 24 hpi, both cells infected with one virus and coinfected cells exhibited the highest titer, surpassing 10^7^ TCID50/mL. However, compared with a single infection of PEDV or PDCoV, the titer of the PEDV virus decreased significantly after co-infection, but the titer of the PDCoV virus did not change significantly (Figure 1E). These findings showed that both viruses may successfully replicate in LLC-PK cells.

### 3.2. Transcriptional Profiles of PEDV-Infected, PDCoV-Infected, and PEDV and PDCoV-Coinfected LLC-PK Cells

**Figure 2:**
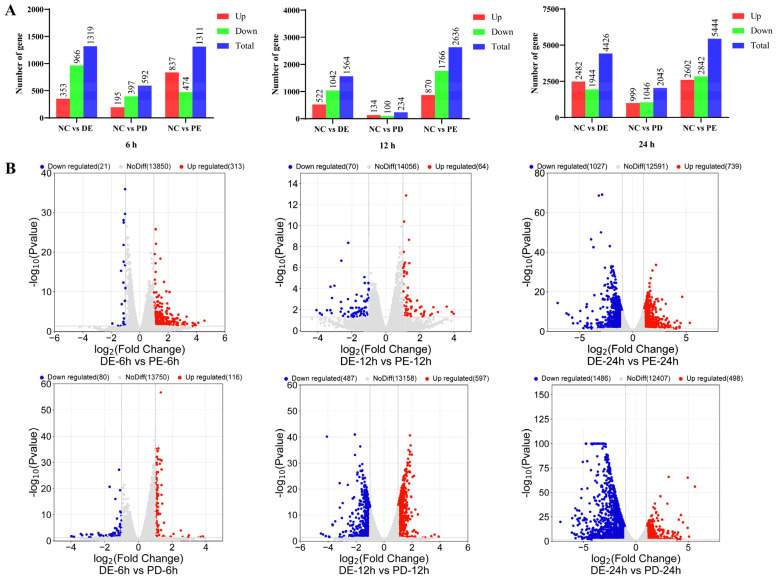
The genes that were differentially expressed in each group. (**A**) The number of DEGs at different time points. (**B**) Volcano plot showing the DEGs. Abbreviations: DE: PDCoV + PEDV; PD: PDCoV; PE: PEDV.

PEDV, PDCoV, and PEDV and PDCoV were used to infect cells, and samples were taken at 0, 6, 12, and 24 h post-infection for whole-genome transcriptome analysis. The transcriptome data revealed 22,519 differentially expressed genes in total, including 10,530 upregulated and 11,989 downregulated genes. At 24 hpi, the total number of DEGs in cells with PEDV, PDCoV, and PEDV and PDCoV infection was 5444, 2045, and 4426, respectively, as shown in Figure 2A. Notably, regardless of whether the cells were infected with a virus or both viruses, the effect of the virus on the cells peaked at 24 hpi, as indicated by the greater numbers of up- and downregulated DEGs at that time point than at 6 and 12 hpi.

According to comparative analysis of the DEGs, the number of DEGs at 24 hpi was greater than that at 6 and 12 hpi in LLC-PK cells coinfected with PEDV and PDCoV. At 24 hpi, compared with PEDV and PDCoV coinfection, PEDV infection alone caused the upregulation of 739 genes and the downregulation of 1027 genes, while PDCoV infection alone caused the upregulation of 498 genes and the downregulation of 1486 genes (Figure 2B).

### 3.3. KEGG Pathway Enrichment Analysis of DEPs between the Coinfection Group and Groups Infected with Individual Viruses

**Figure 3:**
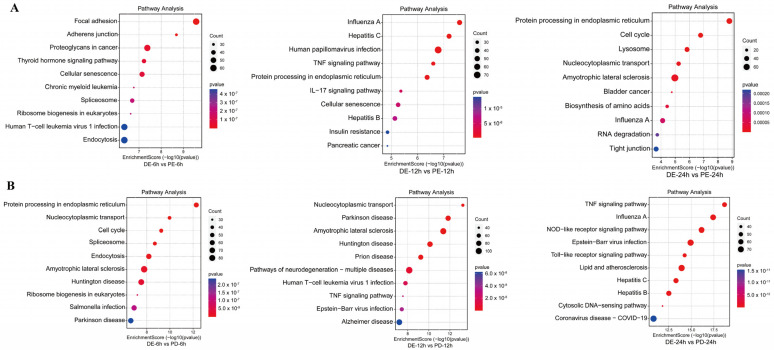
Pathway analysis of DEGs between the coinfection group and groups infected with individual viruses. (**A**) KEGG analysis of DEGs between the PEDV and PDCoV coinfection groups and the PEDV infection group; (**B**) KEGG analysis of DEGs between the PEDV and PDCoV coinfection groups and the PDCoV infection group Abbreviations: DE: PDCoV + PEDV; PD: PDCoV; PE: PEDV.

KEGG enrichment analysis of DEPs between the PEDV and PDCoV coinfection groups and the PEDV infection alone group at different time points showed that at 6 hpi, the DEPs were primarily involved in the cell signaling pathway, ribosome synthesis in eukaryotes, and disease-associated pathways. At 12 hpi, the DEPs were primarily involved in the cytokine signaling pathway, the disease-associated pathway, and the TNF signaling pathway. At 24 hpi, the DEPs were primarily involved in the biosynthesis of amino acids, the p53 signaling pathway, and the cell signaling pathway (Figure 3A).

KEGG enrichment analysis of DEPs between the PEDV and PDCoV coinfection groups and the PDCoV infection group at different time points showed that at 6 hpi, the DEPs were primarily involved in protein processing in the endoplasmic reticulum, ribosome biogenesis in eukaryotes, and disease-associated pathways. At 12 hpi, the DEPs were primarily involved in disease-associated pathways and the TNF signaling pathway. At 24 hpi, the DEPs were involved mainly in the TNF signaling pathway, disease-associated pathway, TNF signaling pathway, NOD-like receptor signaling pathway, Toll-like receptor signaling pathway, RIG-I-like receptor signaling pathway, and NF-kappa B signaling pathway (Figure 3B).

### 3.4. GO and KEGG Pathway Enrichment Analysis of Shared DEGs

**Figure 4:**
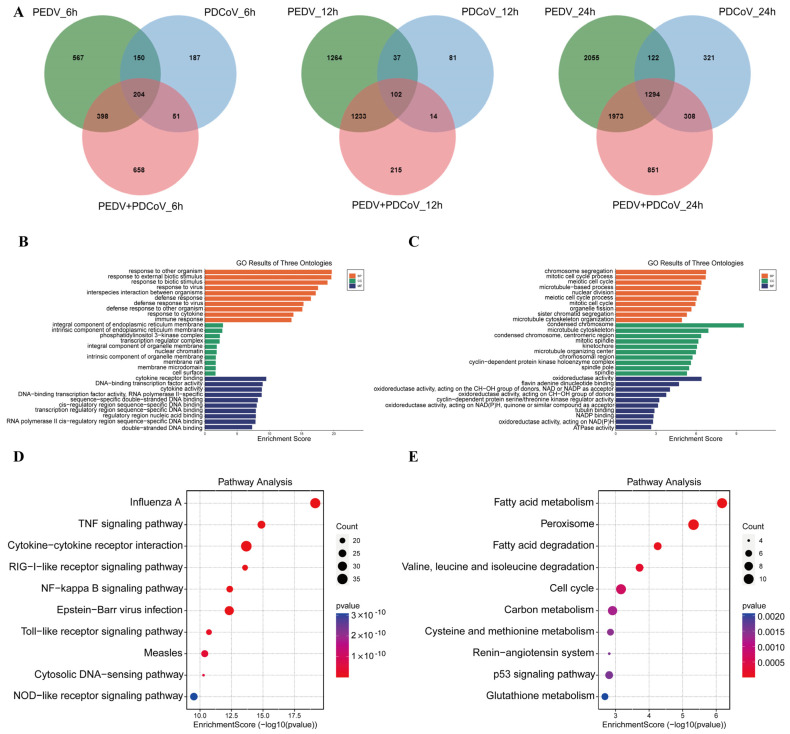
Pathways are associated with the shared DEGs. (**A**) DEGs in the different groups at different time points. (**B**,**C**) GO analysis of the upregulated (**B**) and downregulated (**C**) DEGs. (**D**,**E**) KEGG analysis of the up- (**D**) and down- (**E**) DEGs.

Additionally, a Venn diagram was generated to examine the shared and unique DEGs in the coinfected group. A total of 1294 DEGs were obviously enriched in the PEDV and PDCoV coinfected group at 24 hpi compared with 6 and 12 hpi (Figure 4A). The most DEGs were observed at 24 hpi. Therefore, we examined the biological and molecular functions of the upregulated and downregulated genes, focusing on the shared DEGs among infected cells at 24 hpi.

Possible biological interactions between DEGs were investigated, and GO enrichment analysis was carried out to assess the function of the shared DEGs in the coinfected cells at 24 hpi. GO enrichment was also performed for the 1294 shared DEGs. The upregulated DEGs were mainly enriched in the regulation of response to stimulus, defense response, cellular response to organic substances, cytokine-mediated signaling, and regulation of immune system process pathways (Figure 4B), while the downregulated DEGs were enriched in chromosome segregation, mitotic cell cycle, condensed chromosome, and oxidoreductase activity (Figure 4C). These findings suggest that in the coinfected cells at 24 hpi, the biological mechanism, cell component, and molecular function terms in which the upregulated and downregulated shared DEGs were enriched were distinct.

Moreover, according to KEGG analysis, the upregulated DEGs were enriched mainly in the TNF, RIG-I, NF-kappa B, Toll, and NOD signaling pathways, while the downregulated DEGs were enriched mainly in the metabolism signaling pathway, p53 signaling pathway, and cell cycle (Figure 4D,E). There was inconsistency between the pathways in which the downregulated DEGs and upregulated DEGs were enriched.

### 3.5. Assessment of the Expression of Genes Stimulated by Interferons

ISGs, which are genes whose expression is induced by interferons (IFNs), play important roles in host resistance to virus infection. Studies have shown that ISGs can target different stages of virus replication and inhibit viral infection. As a result, we looked more closely at ISGs, which were among the shared DEGs. It was found that there were 43 ISGs among the upregulated DEGs in cells infected with PDCoV and/or PEDV infection (Table 2).

**Figure 5:**
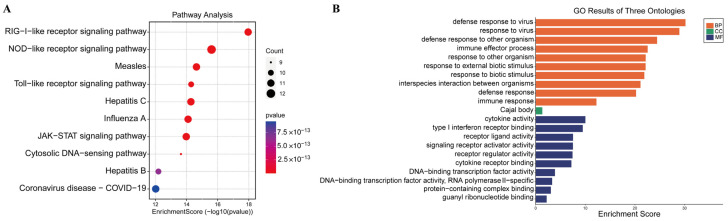
Pathways are associated with the 43 differentially expressed ISGs. (**A**) KEGG analysis; (**B**) GO analysis.

The ISGs were implicated in immune response- and virus infection-related pathways, such as cytosolic DNA-sensing pathways, NOD-like receptor signaling, Toll-like receptor signaling, RIG-I-like receptor signaling, and JAK-STAT signaling, according to KEGG enrichment analysis (Figure 5A).

The enriched genes were mostly involved in immune effector processes, cytokine activity pathways, defense responses to viruses, and defense responses to other organisms, according to GO enrichment analysis (Figure 5B). These findings imply that these ISGs would make good research subjects in future antiviral studies.

### 3.6. Real-Time qPCR Verification of the Differential Expression of ISGs

**Figure 6:**
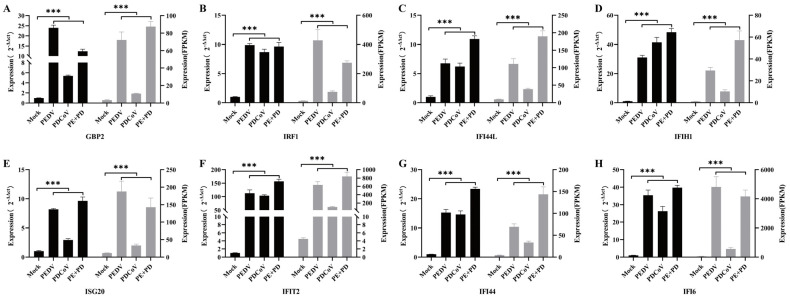
The expression levels of GBP2 (**A**), IRF1 (**B**), IFI44L (**C**), IFIH1 (**D**), ISG20 (**E**), IFIT2 (**F**), IFI44 (**G**) and IFI6 (**H**) were compared at 24 hpi with RT–PCR. Gene expression levels, as measured by qRT–PCR, are plotted on the left axis, while RNA-seq expression levels in FPKM units are plotted on the right axis. mRNA expression levels were normalized to pig ACTB mRNA levels. The data are presented as the means ± SDs; *n* = 3. *** represents *p* < 0.001.

In the present study, eight differentially expressed ISGs were chosen at random, and qRT–PCR were used to assess the changes in their expression to verify the accuracy of the RNA–seq data. Although the fold changes in the expression of these genes revealed by the qRT–PCR and RNA–seq data differed, the results demonstrated that the gene expression changes revealed by qRT–PCR were consistent with those shown by RNA–seq (Figure 6). This difference may have been due to variations in the sensitivity and specificity of qRT–PCR and high-throughput sequencing. According to our findings, the RNA-seq data were largely reliable. The results showed that the expression levels of most ISGs in the co-infection group were significantly higher than those in the single-infection group, indicating that PEDV and PDCoV have a synergistic effect in inducing innate immunity.

### 3.7. Overexpression of ISG20 Inhibits Viral Infection, While Knocking down ISG20 Enhanced Viral Infection

**Figure 7:**
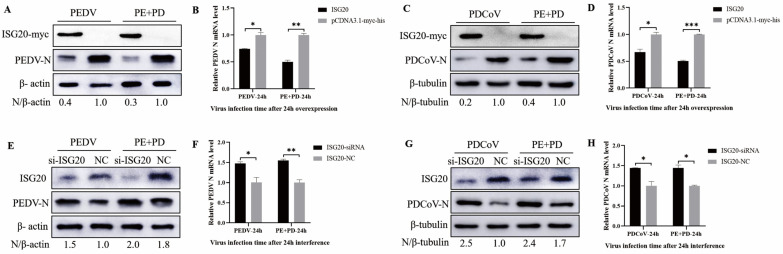
ISG20 inhibits PEDV and PDCoV infection (**A**–**H**). Effect of ISG20 overexpression on infection with PEDV, PDCoV, or PEDV and PDCoV. LLC-PK1 cells were transfected with pCDNA3.1-ISG20-myc or an empty vector for 24 h and then infected with PEDV (MOI = 0.01), PDCoV (MOI = 0.01), or PEDV and PDCoV (MOI = 0.01 for each virus). At 24 hpi, the cells were harvested and subjected to Western blotting (**A**,**C**) and RT–qPCR (**B**,**D**). The impact of ISG20 knockdown on infection with PEDV, PDCoV, or PEDV and PDCoV infection. Following transfection with siRNA targeting ISG20 or control siRNA, LLC-PK1 cells were infected for 24 h with PEDV (MOI = 0.01), PDCoV (MOI = 0.01), or PEDV and PDCoV (MOI = 0.01 for each virus). After the cell samples were collected, they were subjected to Western blotting (**E**,**G**) and RT–qPCR (**F**,**H**) to determine the relative expression of viral mRNA and the ISG20 protein, respectively. The average and standard deviations of the data from two separate experiments are shown. * represents *p* < 0.05, ** represents *p* < 0.01, and *** represents *p* < 0.001.

To assess the role of ISG20 in PEDV, PDCoV, and PEDV + PDCoV infection, two types of experiments were conducted: overexpression and small interfering RNA (siRNA) experiments. First, LLC-PK cells were transfected with pCDNA3.1-ISG20-myc and then infected with PEDV (MOI = 0.01), PDCoV (MOI = 0.01), or PEDV and PDCoV (MOI = 0.01 for each virus) for 24 h. The results showed that overexpression of ISG20 inhibited PEDV and PDCoV infection, as evidenced by decreased N protein expression (Figure 7A,C) and viral mRNA expression (Figure 7B,D). LLC-PK1 cells were transfected with a specific siRNA to inhibit ISG20 expression and subsequently infected with PEDV, PDCoV, or PEDV and PDCoV to further validate the anti-PEDV and anti-PDCoV effects of ISG20. Western blotting (Figure 7E,G) and quantitative real-time RT–PCR (Figure 7F,H) revealed that, compared with the control siRNA, the ISG20-specific siRNA significantly reduced ISG20 expression and enhanced PEDV and PDCoV infection. Taken together, these results suggest that ISG20 has anti-PDCoV and anti-PEDV effects.

## 4. Discussion

Devastating economic losses in the swine industry are caused by the PEDV and PDCoV viruses most commonly found on pig farms in China. Research shows that infection with PEDV and PDCoV activates the JAK-STAT1 signaling pathway and induces ISG expression [33,34]. Furthermore, distinct protein expression patterns were noted in cells infected with both conventional and pandemic PEDV strains. Relevant proteins include RLRs and autophagy-related proteins [35,36]. Reportedly, the replication of PDCoV was significantly inhibited by two innate immunity-related miRNAs, ssc-miR-30c-3p and ssc-miR-374b-3p, in IPEC-J2 cells [37]. However, thus far, there are no published reports on DEGs in LLC-PK cells coinfected with PDCoV and PEDV.

The transcriptomes of cells infected with PEDV and/or PDCoV were compared in this work. Coinfection with PEDV and PDCoV resulted in a greater number of DEGs than infection with either virus alone; these DEGs were primarily associated with the terms organelle portion (CC), immune system process (MF), protein binding (BP), etc. When LLC-PK cells coinfected with PEDV and PDCoV were used as a blank control, the number of DEGs in the PDCoV infection group was smaller than that in the PEDV infection group. The pathways in which the upregulated DEGs were enriched were found to be different than those in which the downregulated DEGs were enriched, according to KEGG analysis. As a result, we decided to further investigate the common DEGs linked to immunological responses.

Viral coinfection usually increases pathogenicity, accelerating the progression of disease and increasing mortality rates. Many studies have shown that common factors regulating viral coinfection, targets of signaling pathways, recombination factors or epigenetic modifications, changes in the microenvironment, and interference with apoptosis all play a key role in increasing pathogenicity [38,39,40,41]. Transcriptome analysis was used to analyze differential gene expression between PEDV and/or PDCoV infection; the DEGs were mainly related to the activation of the host innate immune pathway and apoptosis. In the present study, changes in IFN expression were detected in both the single-infection and coinfection groups. Changes in differential gene expression revealed that the NF-kappa B signaling, RIG-I-like receptor signaling, Toll-like receptor signaling, JAK-STAT signaling, TNF signaling, and NOD-like receptor signaling pathways associated with PEDV and PDCoV were activated. Viral infection causes an innate antiviral immune response and host defense response in LLC-PK cells, which strongly upregulated IFN and ISGs in this study. The specific mechanism underlying the difference in cell expression levels between PEDV-infected and PDCoV-infected cells, between PEDV- or PDCoV-infected cells and coninfected cells, and between coinfected cells and coinfected cells requires further analysis to confirm the relationship between the virus and host.

The transcription of PEDV and PDCoV has been mostly studied in the porcine intestinal epithelial cell line J2 to explore the host response. Researchers have used the porcine intestinal epithelial cell line J2 as a host for infection with the PEDV classical strain and analyzed the cellular gene expression profile [42]. For the first time, to broaden our understanding of PEDV pathogenesis, the transcription profiles of nsp15-transfected IPEC-J2 cells were examined [43]. Researchers have explored the gene expression profiles of PK-15 cells at 0, 24, and 36 hpi post-PDCoV infection by RNA sequencing and found that extensive gene functions and signaling pathways, including innate immune-associated functions and pathways, are affected by PDCoV infection [44]. Studies have shown that PDCoV downregulates many signaling pathways, including the IFN pathway, after infection of porcine intestinal epithelial cells (IPEC-J2) and human intestinal epithelial cells (HIECs) [45]. Transcriptome analysis was used to quantitatively identify DEGs after PDCoV infection in ST cells. PDCoV infection activates the NF-κB signaling pathway and leads to the expression of inflammatory factors, which may be related to TLRs, but TLR2 is not a critical factor [46].

To date, similar studies on LLC-PK cells have not been reported, especially studies on the transcriptome of LLC-PK cells coinfected with PEDV and PDCoV. LLC-PK cells were used for the first time as a model for transcriptome analysis following PEDV and/or PDCoV infection, which provided reference data for better understanding the specific pathogenesis of PEDV and PDCoV coinfection.

Different viral infections can lead to changes in the expression of various genes in the host, so that the host presents different physiological states. After the virus invades the body, it stimulates the body’s cells to produce hundreds of ISGs by inducing type I and III IFNs, thereby exerting an antiviral effect. The proteins expressed by ISGs can not only directly inhibit viral replication but also indirectly exert antiviral effects by regulating the expression of IFNs [47]. The proteins expressed by ISGs cover a wide range of species and have a variety of biological activities, so it is difficult to classify them comprehensively by structure or function. The replication cycle of the virus mainly includes the stages of invasion, shelling, replication, assembly, and release. Current studies have shown that ISGs can effectively target each stage of the virus replication cycle and inhibit its replication, thereby achieving antiviral effects. The study of ISGs not only helps to understand the antiviral mechanism of the host but also provides a theoretical basis and reference for opening up new antiviral strategies and developing new methods for preventing and treating viral diseases. In order to study the expression changes of ISGs in the co-infection group and the difference between the co-infection group and the single infection group.

IFN-1s play a key role in the host immune response as antiviral cytokines. However, depending on the features of the intestinal mucosal surface and microbiota, intestinal coronaviruses elicit distinct responses in intestinal epithelial cells [48]. Type III interferon (IFN-γ) plays a vital role in preventing infection by intestinal coronavirus [49]. According to previous research, PEDV can evade the IFN-γ response in intestinal epithelial cells, suppress IFN-γ production, and interfere with interferon regulatory factor 1 (IRF1) signaling, which prevents IRF1-mediated type III IFN production [48,50]. The activation of the IFN-λ1 promoter but not TBK1 or IRF1 by RIG-I, MDA5, and MAVS is hindered by PDCoV infection [51].

ISG20 was discovered in 1997 in Daudi cells as a new interferon-regulated protein [52]. The expression of this protein can be induced by type I, type II, and type III interferons, among which type I and type II interferons are the main inducers, and poly (IC) can also induce ISG20 expression [53]. ISG20 is a relatively understudied antiviral protein capable of inhibiting a broad spectrum of viruses. At present, it is believed that the antiviral mechanism of ISG20 involves its 3′-5′ exonuclease activity, which results in the degradation of viral RNA. It is believed that through this activity, ISG20 degrades viral RNA, mRNA, or replicons to exert antiviral effects.

Although ISG20 is an important antiviral molecule, exogenous overexpression of ISG20 is detrimental to cell survival; therefore, it is speculated that the expression of ISG20 is strictly regulated in cells [54]. Data from the ISG20 overexpression and interference experiments have shown that ISG20 has inhibitory effects on single-stranded positive-strand RNA viruses such as Sindbisvirus (SINV), Flaviviridae viruses (West Nile virus, hepatitis C virus, bovine viral diarrhea virus), and Picornaviridae viruses (hepatitis A virus) and has inhibitory effects on single-stranded negative-strand RNA viruses. It also has inhibitory effects on vesicular stomatitis virus (VSV) of the Rhabdoviridae family, influenza A virus of the Orthomyxoviridae family, and bunyavirus. In addition, ISG20 can inhibit the replication of HIV-1 and several DNA viruses, such as the hepatitis B virus. However, ISG20 did not show inhibitory effects on single-strand positive-strand RNA viruses, SARS-CoV, or adenovirus [55]. Although it is rarely mentioned, high levels of ISG20 expression lead to cell death, and it is currently unknown whether regulatory mechanisms exist to prevent this deleterious effect [54,56].

We further assessed the antiviral efficacy of ISG20 in cells infected with PDCoV and PEDV. In LLC-PK cells, overexpression of ISG20 dramatically inhibited PEDV and PDCoV infection, and ISG20 significantly inhibited PEDV and PDCoV coninfection in LLC-PK cells. Furthermore, in LLC-PK cells, the knockdown of ISG20 by siRNA significantly promoted PEDV or PDCoV infection and co-infection of PEDV and PDCoV (Figure 7). However, the difference between the ability of ISG20 to inhibit infection with PEDV or PDCoV and that of ISG20 to inhibit PEDV and PDCoV coinfection could not be proven. As an exonuclease, the antiviral function of ISG20 is mostly related to its exonuclease activity. For example, ISG20 can interact with BTV VP4 protein, and the interaction between ISG20 and VP4 is significantly weakened without exonuclease activity, and the inhibitory effect on BTV is also weakened, indicating that the exonuclease activity of ISG20 is an important factor inhibiting BTV replication [57]. Qu et al. found that ISG20 colocalizes with IAV NP and interacts with IAV NP to inhibit IAV replication, which depends on its exonuclease activity [58]. Overexpression of ISG20 in HeLa cells showed antiviral activity against three RNA viruses: vesicular stomatitis virus (VSV), influenza virus, and encephalomyocarditis virus. Further studies showed that overexpression of the exonuclease-activity-deficient ISG20 protein in cells had no antiviral effect. It is demonstrated that the antiviral effect depends on its exonuclease activity [59]. So, whether the antiviral effect of ISG20 on PEDV and PDCoV also depends on its exonuclease activity and whether ISG20 has the same antiviral mechanism for PEDV or PDCoV, these need further research, which is also the direction of our follow-up efforts.

In general, ISG20 is an important antiviral effector involved in the innate immune response. Therefore, elucidating the molecular mechanism underlying its effect in cells may aid in the development of new antiviral therapies and provide clues about other cellular processes of wider interest.

## 5. Conclusions

In summary, in this study, the transcriptomes of LLC-PK cells infected with PEDV and/or PDCoV were first compared. Changes in IFN expression were found in response to PEDV or PDCoV infection and PEDV and PDCoV coinfection through differential gene expression analysis and functional enrichment analysis. The findings demonstrated that the transcriptomes of the cells differed, particularly those shared by DEGs and ISGs. This demonstrated that there are variations in the responses of cells infected with PDCoV and/or PEDV. We discovered that ISG20 inhibits both PEDV infection and PDCoV infection, demonstrating that ISG20 is a significant antiviral ISG. To fully elucidate the molecular mechanism and antiviral activity of ISG20, additional research is needed. The information from this study is helpful for better understanding the host response to coinfection with PEDV and PDCoV, laying a foundation for further study of the pathogenic mechanism of coinfection with porcine intestinal coronaviruses.

## Figures and Tables

**Table 1:** The primers used for qRT–PCR in this study.

Genes	Forward Primer	Reverse Primer	Amplicon Size (bp)
*GBP2*	GCAGCTACCGGAAGAAGATG	TTGACAAAGGTCAGCACCAG	153
*IRF1*	AAGTCCAGCCGAGATGCTAA	GGCCTGTTCAATGTCCAAGT	162
*IFI44L*	GGCCATAGTGGGCTCTGATA	CTGTGATTCAGCGATGGAGA	231
*IFIH1*	CTCAAAGAGCATCCCCTGAG	GTTCGAACTCTTTGCGGAAG	246
*ISG20*	AGATCCTGCAGCTCCTGAAA	CCAACACACTGTCCTGGATG	216
*IFIT2*	CCTGGGAAGACTTGCAGAAG	GGCCAGTTATCCAGACGGTA	249
*IFI44*	GAGGCCTGGTTCACCAAATA	GGCACAGTCTGCCTTCTTTC	249
*IFI6*	CCAAAACGAAACGCAATACA	CTCCACCGCAGGTGTAGAGT	163
*β-Actin*	CTTCCTGGGCATGGAGTCC	GGCGCGATGATCTTGATCTTC	201

**Table 2:** Antiviral interferon-stimulated genes in this study.

Gene ID	PEDV Infection		PDCoV Infection		PEDV + PDCoV Infection		Description
	log2 Fold Change	*p* Values	log2 Fold Change	*p* Values	log2 Fold Change	*p* Values	
ENSSSCG00000002002	6.310629225	6.20 × 10^−11^	5.494214783	1.66 × 10^−7^	6.767597361	1.27 × 10^−15^	interferon regulatory factor 9
ENSSSCG00000003091	1.234811026	7.39 × 10^−12^	– ^a^	–	1.401705148	1.30 × 10^−14^	interferon regulatory factor 2 binding protein 1
ENSSSCG00000003178	1.685537515	1.08 × 10^−21^	–	–	1.342602373	7.28 × 10^−19^	interferon regulatory factor 3
ENSSSCG00000003763	4.008604395	3.69 × 10^−100^	2.809789843	2.44 × 10^−84^	4.972617711	9.57 × 10^−107^	interferon induced protein 44
ENSSSCG00000005136	1.089794081	7.66 × 10^−3^	–	–	–	–	interferon epsilon
ENSSSCG00000005163	1.479543723	9.92 × 10^−38^	1.257973246	2.68 × 10^−13^	1.887533485	6.16 × 10^−18^	interferon beta 1
ENSSSCG00000006923	4.468281465	4.91 × 10^−71^	1.575633517	1.79 × 10^−26^	4.658103435	5.66 × 10^−188^	guanylate binding protein 2, interferon-inducible
ENSSSCG00000010451	7.219000344	1.99 × 10^−135^	4.494882714	7.29 × 10^−103^	7.526928847	1.71 × 10^−221^	interferon induced protein with tetratricopeptide repeats 2
ENSSSCG00000010452	6.567469339	5.33 × 10^−229^	4.725627596	7.70 × 10^−53^	7.425033228	1.81 × 10^−114^	interferon-induced protein with tetratricopeptide repeats 1
ENSSSCG00000010454	1.739174062	2.76 × 10^−24^	–	–	2.829380505	8.35 × 10^−82^	interferon-induced protein with tetratricopeptide repeats 5
ENSSSCG00000011402	1.161609759	9.63 × 10^−11^	–	–	–	–	interferon related developmental regulator 2
ENSSSCG00000012076	7.31026276	7.83 × 10^−51^	4.816830348	6.30 × 10^−128^	7.525711196	6.16 × 10^−38^	Interferon-induced GTP-binding protein Mx2
ENSSSCG00000012853	6.744825817	5.60 × 10^−37^	3.394978684	2.39 × 10^−55^	6.294980934	1.30 × 10^−76^	interferon regulatory factor 7
ENSSSCG00000014277	5.1491548	9.84 × 10^−50^	2.465843809	8.59 × 10^−74^	4.412829187	3.74 × 10^−181^	interferon regulatory factor 1
ENSSSCG00000014349	3.849359797	1.34 × 10^−16^	–	–	2.028133067	3.05 × 10^−28^	stimulator of interferon response cGAMP interactor 1
ENSSSCG00000014565	3.847166684	1.17 × 10^−36^	–	–	2.813431636	2.94 × 10^−90^	interferon-induced transmembrane protein 1
ENSSSCG00000015612	1.341585174	5.57 × 10^−12^	–	–	–	–	interferon regulatory factor 6
ENSSSCG00000015782	3.485083398	1.51 × 10^−35^	1.173186109	2.19 × 10^−14^	3.497169917	5.54 × 10^−109^	interferon regulatory factor 2
ENSSSCG00000015897	4.757076534	7.59 × 10^−125^	3.087070238	2.77 × 10^−49^	5.62226484	1.45 × 10^−99^	interferon induced with helicase C domain 1
ENSSSCG00000016573	3.00658889	1.38 × 10^−12^	1.889624403	8.31 × 10^−16^	3.031780305	6.00 × 10^−42^	interferon regulatory factor 5
ENSSSCG00000021504	10.66478654	7.55 × 10^−13^	8.54769866	4.67 × 10^−36^	9.668453582	2.20 × 10^−9^	interferon-alphaomega
ENSSSCG00000024867	4.121896082	3.24 × 10^−43^	1.47607923	1.39 × 10^−26^	3.640161424	1.43 × 10^−27^	interferon stimulated exonuclease gene 20
ENSSSCG00000024973	6.542282639	7.60 × 10^−71^	2.506733001	1.20 × 10^−61^	5.723463173	1.22 × 10^−257^	guanylate binding protein 1, interferon-inducible
ENSSSCG00000027660	3.578893585	1.57 × 10^−45^	1.917801061	3.74 × 10^−47^	4.265499699	7.20 × 10^−173^	interferon induced protein 44 like
ENSSSCG00000029763	2.447517449	9.82 × 10^−44^	–	–	1.746768492	2.81 × 10^−31^	interferon induced protein 35
ENSSSCG00000031149	2.654658654	4.72 × 10^−19^	–	–	2.754558433	1.63 × 10^−5^	interferon-alpha-15
ENSSSCG00000031529	9.039504874	8.45 × 10^−92^	2.829509587	2.93 × 10^−3^	8.194740952	3.70 × 10^−35^	interferon omega 1
ENSSSCG00000032356	1.356456477	1.41 × 10^−25^	1.256657445	7.59 × 10^−4^	1.634339027	3.09 × 10^−19^	interferon lambda-4
ENSSSCG00000032591	1.069077146	2.03 × 10^−9^	–	–	–	–	interferon-induced transmembrane protein 1-like
ENSSSCG00000033089	9.881950086	6.94 × 10^−73^	5.947358031	1.25 × 10^−32^	9.656541338	1.04 × 10^−199^	interferon-induced very large GTPase 1-like
ENSSSCG00000033115	–	–	–	–	1.643246776	3.95 × 10^−28^	interferon alpha and beta receptor subunit 1
ENSSSCG00000034570	6.605547683	9.58 × 10^−93^	3.338971302	2.86 × 10^−29^	6.307464556	1.72 × 10^−134^	interferon alpha inducible protein 6
ENSSSCG00000034980	7.781934489	1.95 × 10^−18^	3.806621518	3.53 × 10^−4^	6.767895161	5.66 × 10^−29^	interferon regulatory factor 8
ENSSSCG00000035185	1.388993434	3.10 × 10^−25^	1.236766902	2.84 × 10^−6^	1.323690765	1.45 × 10^−10^	interferon lambda-3-like
ENSSSCG00000036439	10.59936502	2.23 × 10^−53^	7.451354106	1.02 × 10^−57^	10.6376492	4.52 × 10^−123^	interleukin 29 (interferon, lambda 1)
ENSSSCG00000036556	2.721750164	1.37 × 10^−48^	–	–	2.818164244	1.79 × 10^−71^	interferon alpha and beta receptor subunit 2
ENSSSCG00000038132	1.559518738	3.75 × 10^−20^	–	–	2.022454176	2.29 × 10^−46^	interferon regulatory factor 2 binding protein 2
ENSSSCG00000038912	2.834263813	8.28 × 10^−29^	–	–	2.48353821	1.41 × 10^−46^	interferon induced transmembrane protein 3
ENSSSCG00000040814	1.599755345	3.76 × 10^−21^	1.387675563	3.19 × 10^−4^	1.597754332	2.26 × 10^−6^	interleukin 28B (interferon, lambda 3)
ENSSSCG00000043301	2.108776423	3.21 × 10^−4^	2.386654345	4.87 × 10^−2^	2.386454745	5.77 × 10^−6^	interferon-alpha-8
ENSSSCG00000044440	1.389538790	5.86 × 10^−13^	–	–	1.385354889	3.18 × 10^−8^	interferon-alpha-4
ENSSSCG00000045163	1.298445331	2.83 × 10^−21^	–	–	1.534679975	1.43 × 10^−9^	interferon-alpha-9
ENSSSCG00000050619	3.678654549	6.64 × 10^−46^	2.66908943	1.03 × 10^−2^	3.598458533	8.29 × 10^−34^	Sus scrofa interferon, alpha 1 (IFNA1), mRNA.

^a^ Genes with no differential expression are indicated by ‘–’.

## Data Availability

The raw data supporting the conclusions of this article will be made available by the authors without undue reservation to any qualified researcher.
